# Alterations of the fecal and vaginal microbiomes in patients with systemic lupus erythematosus and their associations with immunological profiles

**DOI:** 10.3389/fimmu.2023.1135861

**Published:** 2023-03-10

**Authors:** Zongxin Ling, Yiwen Cheng, Jie Gao, Wenhui Lei, Xiumei Yan, Xiaogang Hu, Li Shao, Xia Liu, Runfang Kang

**Affiliations:** ^1^ Collaborative Innovation Center for Diagnosis and Treatment of Infectious Diseases, State Key Laboratory for Diagnosis and Treatment of Infectious Diseases, National Clinical Research Center for Infectious Diseases, The First Affiliated Hospital, School of Medicine, Zhejiang University, Hangzhou, Zhejiang, China; ^2^ Jinan Microecological Biomedicine Shandong Laboratory, Jinan, Shandong, China; ^3^ Department of Laboratory Medicine, Shandong Provincial Hospital Affiliated to Shandong First Medical University, Jinan, Shandong, China; ^4^ Department of Geriatrics, Lishui Second People’s Hospital, Lishui, Zhejiang, China; ^5^ School of Clinical Medicine, Institute of Hepatology and Metabolic Diseases, Hangzhou Normal University, The Affiliated Hospital of Hangzhou Normal University, Hangzhou, Zhejiang, China; ^6^ Department of Intensive Care Unit, The First Affiliated Hospital, School of Medicine, Zhejiang University, Hangzhou, Zhejiang, China; ^7^ Department of Dermatology, Lishui Second People’s Hospital, Lishui, Zhejiang, China

**Keywords:** feces, immunology, microbiome, systemic lupus erythematosus, vagina

## Abstract

**Background:**

Exploring the human microbiome in multiple body niches is beneficial for clinicians to determine which microbial dysbiosis should be targeted first. We aimed to study whether both the fecal and vaginal microbiomes are disrupted in SLE patients and whether they are correlated, as well as their associations with immunological features.

**Methods:**

A group of 30 SLE patients and 30 BMI-age-matched healthy controls were recruited. Fecal and vaginal samples were collected, the 16S rRNA gene was sequenced to profile microbiomes, and immunological features were examined.

**Results:**

Distinct fecal and vaginal bacterial communities and decreased microbial diversity in feces compared with the vagina were found in SLE patients and controls. Altered bacterial communities were found in the feces and vaginas of patients. Compared with the controls, the SLE group had slightly lower gut bacterial diversity, which was accompanied by significantly higher bacterial diversity in their vaginas. The most predominant bacteria differed between feces and the vagina in all groups. Eleven genera differed in patients’ feces; for example, *Gardnerella* and *Lactobacillus* increased, whereas *Faecalibacterium* decreased. Almost all the 13 genera differed in SLE patients’ vaginas, showing higher abundances except for *Lactobacillus*. Three genera in feces and 11 genera in the vagina were biomarkers for SLE patients. The distinct immunological features were only associated with patients’ vaginal microbiomes; for example, *Escherichia−Shigella* was negatively associated with serum C4.

**Conclusions:**

Although SLE patients had fecal and vaginal dysbiosis, dysbiosis in the vagina was more obvious than that in feces. Additionally, only the vaginal microbiome interacted with patients’ immunological features.

## Introduction

Systemic lupus erythematosus (SLE) is an autoimmune disease characterized by the overactivation of the immune system and the involvement of various organs. The global systemic lupus erythematous (SLE) incidence and newly diagnosed population were estimated to be 5.14 (1.4 to 15.13) per 100,000 person-years and 0.40 million people annually, respectively (1). Mortality from SLE is two to three times higher than that of the general population (2) and was the underlying cause of over 50,000 deaths from 1968 to 2012 (3). SLE results from a complex interplay of multiple genetic and environmental exposure (4); however, much of the etiology of SLE remains unknown.

Throughout our lives, we are immersed in and colonized by immense and complex microbial communities. These microbiomes are essential for immune homeostasis. Mounting evidence supports the notion that dysbiosis in the blood (5), gut (6–13), oral cavity (10, 14–17), skin (18), and bladder (19) is associated with SLE. It is worth noting that most previous studies have reported gut dysbiosis in SLE patients, but several have demonstrated that a balanced gut microbiome is accompanied by an imbalanced microbiome in other body niches, such as the blood (5) and oral cavity (16), when the same cohort of SLE patients and healthy controls (HC) were involved in the studies. These findings suggest that the onset of dysbiosis in other body niches occurs before gut dysbiosis. The identification of the primary dysbiosis in various body niches might be critical as it serves as an indicator that dysbiosis needs to be modulated.

A previous study reported that dysbiosis in the vagina is evident in patients with primary Sjögren’s syndrome (PSS) (20), a systemic inflammatory autoimmune disease that shares epidemiological, clinical, pathogenic, and etiological features with SLE (21, 22). In addition, women are more frequently affected by SLE than men (1). Therefore, we questioned whether there might be a distinct vaginal microbiome signature of SLE in women. However, no study to date has explicitly assessed the relationship between the vaginal microbiome and SLE.

Several immune disorders are associated with SLE. For example, the complement system involves immune systems and has important roles in the pathogenesis of SLE (23). The association between complement deficiencies are associated with microbial dysbiosis (24). Like complements, low levels of immunoglobulins can be a critical issue in SLE (25). Previous studies reported that the disorders of complement and immunoglobulins in SLE play a role in gut and skin dysbiosis (10, 24, 26). However, no report has revealed the associations between immunological profiles and microbiomes in the gut and vagina from the same cohort of SLE patients. The aim of our study was to assess whether SLE is associated with a disease-specific microbiome composition in the gut and the vagina, and their associations with immunological profiles in patients.

## Methods and materials

### Enrollment of study subjects

We performed a cross-sectional study consisting of 30 SLE premenopausal females and 30 age-BMI-matched premenopausal healthy controls (HC). Samples were obtained from the Department of Dermatology, Lishui Second People’s Hospital (Zhejiang, China) and the First Affiliated Hospital, School of Medicine, Zhejiang University. The criteria for inclusion were an age of ≥18 years and a willingness to participate in the present study. The criteria for exclusion were individuals who were pregnant or breastfeeding, had severe illnesses, such as tumors and infections (e.g., enteritis, vaginitis, and urinary tract infection), and had received antibiotics and probiotics within the previous 30 days.

### Sample collection

Fecal material was collected in a container by the participants, and 30 mg was immediately placed in a sterile container. The vaginal sample was collected by a nurse using a sterile DNA-free swab inserted into the middle section of the vaginal tract. All samples were placed in sterile DNA- and enzyme-free centrifuge tubes and immediately stored at −80°C until use.

Blood samples were collected on the day when the participants were recruited. An immunoturbidimetric test was used to assess the serum immunological profiles, such as complement (C) 3, C4, IgA, IgG, and IgM in the blood (AU5421; Beckman Coulter).

### DNA extraction and sequencing

Sera-Mag SpeedBead Carboxylate-Modified Magnetic Particles (GE Healthcare UK, Little Chalfont, UK) were used to extract DNA from the fecal and vaginal samples. The quantity and quality of the extracted DNA were measured using a Nanodrop ND-1000 spectrophotometer (Thermo Fisher Scientific, Waltham, MA, USA) and agarose gel electrophoresis, respectively. Polymerase chain reaction (PCR) amplification of the bacterial 16S rRNA gene V3-V4 region was performed using the universal primers 319F and 806R with 30 cycles. PCR amplicons were purified using Agincourt AM Pure XP Beads (Beckman Coulter, Indianapolis, IN, USA) and quantified using a Pico Green dsDNA Assay Kit (Invitrogen, Carlsbad, CA, USA). Following the individual quantification step, amplicons were pooled in equal amounts, and pair-end 2×300 bp sequencing was performed using the Ill lumina MiSeq platform.

### Bioinformatic and statistical analysis

Raw reads of the 16S rRNA gene sequences were trimmed using Cutadapt (cutadapt.readthedocs.io) to remove barcodes and adaptors. The overlapping paired-end reads were merged into a longer tag using FLASH (v1.2.8). Reads were quality trimmed using fd trim (v0.94) from the 3′ end to remove bases with low-quality scores. Reads shorter than 100 bp, with more than 5% Ns, or with an average quality below 20 were discarded. Chimeras were removed using V search (v2.3.4). QIIME2 was used to process the clean reads to generate an ASV table, and the taxonomy of microbes was identified using the Silva database (v138).

R (version 3.6.2) was used for statistics. Beta diversity analysis was performed to evaluate differences in species complexity between samples. We applied the permutational multivariate analysis of the variance method to the Bray–Curtis distance data using 999 permutations to analyze feature differences between patients with SLE and HCs; statistical significance was defined as p<0.05 (R software vegan package). Alpha diversity was calculated based on a rarefied feature table (rarefied at the lowest sample size) and indices of Chao 1, Shannon, and Simpson’s were used. Based on the feature abundances, an UpSet diagram was used to display the numbers of microbial features shared by the various groups. To look for potential biomarkers that could distinguish SLE from controls, we performed classical receiver operating characteristic (ROC) curve analysis. The ROC curve is a plot of sensitivity versus 1−specificity (often called the false-positive rate) that offers a summary of sensitivity and specificity across a range of cut points for a continuous predictor. The area under the curve, or statistic, ranges from 0.5 (no discrimination) to a theoretical maximum of 1 (27). The area under the ROC curve (AUC) results were considered excellent for AUC values between 0.9 and 1, good for AUC values between 0.8 and 0.9, fair for AUC values between 0.7 and 0.8, poor for AUC values between 0.6 and 0.7, and failed for AUC values between 0.5 and 0.6 (28–30). Fisher’s exact test or Student’s t test were applied using SPSS version 27.0 (IBM Corp., Armonk, NY, USA) to compare the clinical variables between the SLE and HC groups. The Wilcoxon rank-sum test was used to compare alpha diversity indices and bacterial abundances, and a Benjamini–Hochberg false discovery rate (FDR) correct was calculated for comparative tests. An FDR of <0.05 was used as a cutoff for comparative statistical tests. Pearson’s correlation analysis was used to assess the correlations between the relative abundances of bacterial genera and immunological profiles in the SLE samples; correlations with p<0.05 were considered significant.

## Results

### Participants’ characteristics

We collected fecal and vaginal samples from 30 SLE women and 30 age-BMI matched controls. Compared with the HC group, the SLE group had a higher level of serum IgG but lower levels of serum C3 and C4 (P<0.05; **Table 1**). As more than half of the patients were administered azathioprine (50.00%), hydroxychloroquine (76.67%), and prednisolone (73.33%) (**Table 1**), we considered them as confounding factors in downstream microbiome analyses. The characteristics and medication usages are listed in **Table S1**.

**Table 1:** Demographics of subjects.

Characteristics	SLE (n=30)	HC (n=30)	P value
Age (years)	39.63 ± 11.55	40.33 ± 11.60	0.431
Body mass index (kg/m^2^)	21.90 ± 3.30	23.44 ± 2.88	0.055
Cause of menopause, n(%)			0.182
Natural menopause	3 (10.00)	5 (100.00)	
Treatment associated	3 (10.00)	0 (0.00)	
Duration of menopause prior to enrollment (yrs)	4.10 ± 2.15	3.85 ± 1.19	0.069
Vaginal pH	5.51 ± 3.00	5.42 ± 2.29	0.090
Duration of SLE (yrs)	6.35 ± 4.37	NA	NA
SLEDAI	7.75 ± 2.54	NA	NA
≥8	25 (83.33)	NA	NA
<8	5(16.67)	NA	NA
Smoking history, n(%)			1.000
Never smoked	30 (100.00)	30 (100.00)	
Former smoker	0 (0.00)	0 (0.00)	
Current smoker	0 (0.00)	0 (0.00)	
Drinking history, n(%)			0.492
Never drunk	28 (93.33)	30 (100.00)	
Former drinker	2 (6.67)	0 (0.00)	
Current drinker	0 (0.00)	0 (0.00)	
Immunological profiles			
ASO positive (U/ml), n(%)	1 (3.33)	0 (0.00)	1.000
Ig A (g/L)	2.30 ± 1.17	2.47 ± 0.15	0.516
Ig G (g/L)	16.54 ± 11.04	10.90 ± 2.34	0.018
Ig M (g/L)	1.07 ± 0.61	1.17 ± 0.94	0.624
C3 (g/L)	0.74 ± 0.23	1.03 ± 0.19	<0.001
C4 (g/L)	0.15 ± 0.06	0.23 ± 0.18	0.029
Medical history			
Diabetes, n(%)	2 (6.67)	3 (10.00)	0.613
Hypertension, n(%)	2 (6.67)	3 (10.00)	0.640
Medicine usages			
Immunosuppressive agents, n(%)			
Azathioprine	15 (50.00)	NA	NA
Ciclosporin A	2 (6.67)	NA	NA
Cyclophosphamide	2 (6.67)	NA	NA
Everolimus	1 (3.33)	NA	NA
Hydroxychloroquine	23 (76.67)	NA	NA
Leflunomide	3 (10.00)	NA	NA
Prednisolone	22 (73.33)	NA	NA
Sulfasalazine	1 (3.33)	NA	NA
Tacrolimus	2 (6.67)	NA	NA

Pearson Chi-square or Fisher’s exact test was used with categorical variables; Student’s t test was used with normalized continuous variables.

ASO, antistreptolysin O; C, complement; Ig, immunoglobulin; LN, lupus nephritis; NA, not applicable; SLEDAI, systemic lupus erythematosus disease activity index.

### The fecal and vaginal microbiomes differed in SLE patients

**Figure 1:**
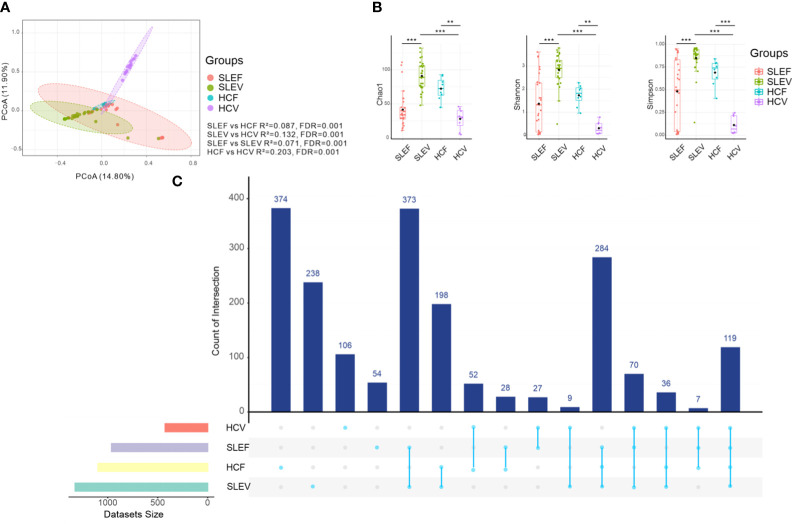
Bacterial composition, bacterial diversity, and UpSet in the feces and vaginas of SLE patients and HC. **(A)** PCoA based on Bray−Curtis distances ASV level. The 95% confidence ellipse is drawn for each group. Permutational multivariate analysis of variance (PERMANOVA) was performed for statistical comparisons of samples in the two groups. The *P* value was adjusted by the Benjamini and Hochberg false discovery rate (FDR). **(B)** Bacterial richness and diversity measured using the Chao 1, Shannon, and Simpson indexes were calculated at the ASV level. The Wilcoxon rank-sum test was performed and adjusted by Benjamini and Hochberg’s false discovery rate (FDR). **P_adj_ < 0.01, ***P_adj_ *<* 0.001. **(C)** UpSet plots illustrating the quantitative intersection of the sets of ASVs across the samples. The numbers above the bars show the number of common ASVs between the groups of the samples of SLEF, SLEV, HCF, and HCV. HCF, HC feces; HCV, HC vagina; SLEF, systemic lupus erythematosus feces; SLEV, systemic lupus erythematosus vagina.

When comparing SLE patients’ fecal samples (SLEF) to HC fecal samples (HCF), we found that the fecal microbiome differed (R^2^ = 0.087, FDR=0.001; **Figure 1A**). Azathioprine, hydroxychloroquine, and prednisolone were not confounding factors, as fecal microbiomes of the medication users and non-users did not differ (FDR >0.05; **Figure S1A–C**). The microbial communities of SLE patients’ vaginal samples (SLEV) and HC vaginal samples (HCVF) were significantly different (R^2^ = 0.132, FDR=0.001; **Figure 1A**) and not affected by medication (FDR>0.05; **Figure S1A–C**). In addition, the SLE and HC groups had a distinct fecal microbiome from their vaginal microbiome (R^2^ = 0.071 [FDR=0.001], and R^2^ = 0.203 [FDR=0.001], respectively; **Figure 1A**).

The fecal microbiome of SLE patients was slightly less rich (Chao 1) than that of its respective HCF (FDR=0.089; **Figure 1B**), whereas the vaginal microbiome of SLE patients was significantly richer than its respective HCV (FDR <0.001; **Figure 1B**). Similarly, reduced bacterial diversity estimators of the Shannon index and Simpson index were only observed in the patients’ vaginal samples, instead of fecal samples, compared with those of the controls (FDR <0.001; **Figure 1B**). For the SLE patients, fecal samples had significantly lower levels of bacterial richness and bacterial diversity than those in the SLEV. However, for the control samples, HCF samples had higher levels of bacterial richness and diversity than those in the HCV samples (FDR <0.01, **Figure 1B**).

The gap between the microbiome in SLE patients and controls was also reflected in the numbers of shared ASVs in the fecal and vaginal samples. For example, the shared number of ASVs of SLEF and HCF was 28, whereas the shared number of ASVs of SLEV and HCV was only 9 (**Figure 1C**), indicating that the gap between SLEF and HCF was smaller than that between SLEV and HCV.

### Bacterial compositions differed in SLE patients

**Figure 2:**
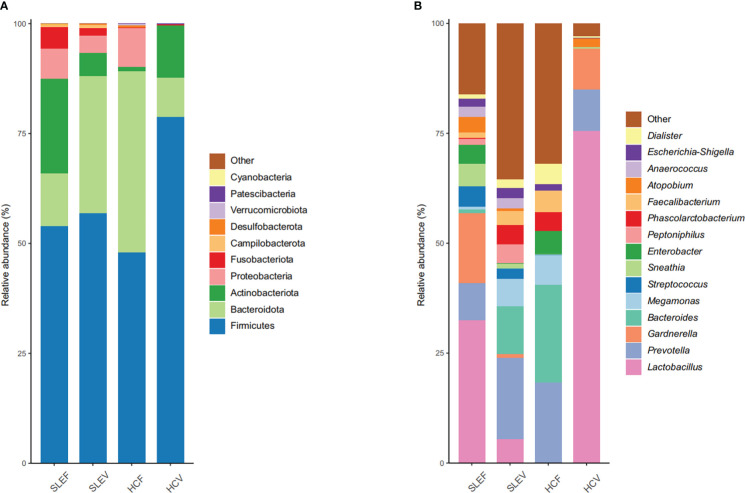
Microbial profile at the phylum **(A)** and genus **(B)** level. Only the top 10 most abundant phyla and 15 most abundant genera are shown. HCF, HC feces; HCV, HC vagina; SLEF, systemic lupus erythematosus feces; SLEV, systemic lupus erythematosus vagina.

Five bacterial phyla, Firmicutes (53.93%), Actinobacteria (21.49%), Bacteroidetes (11.99%), Proteobacteria (6.90%), and Fusobacteria (4.85%), accounted for more than 1% of the total abundance in the SLEF, while three bacterial phyla, Firmicutes (47.91%), Bacteroidota (41.30%), and Proteobacteria (8.81%), accounted for more than 1% of the total of abundance in the HCF samples, (**Figure 2A**). Similar to the comparison between SLEF and HCF, the number of bacterial phyla accounting for more than 1% of the total abundance in SLEV was more than that in HCV: five bacterial phyla, Firmicutes (56.84%), Bacteroidota (31.21%), Actinobacteria (5.30%), Proteobacteria (3.87%), and Fusobacteria (1.69%), were predominant. For the HCV group, three bacterial phyla, Firmicutes (78.73%), Actinobacteria (11.80%), Bacteroidota (8.98%), accounted for more than 1% of the total abundance (**Figure 2A**).

At the bacterial genus level, the top five most abundant bacteria in the SLEF were *Lactobacillus* (32.53%), *Gardnerella* (15.95%), *Prevotella* (8.33%), *Sneathia* (5.13%), and *Streptococcus* (4.70%), while the dominated bacterial genera in the SLEF samples were not the dominant bacteria in the HCF samples, except for *Prevotella* (18.22%). Most of the bacterial sequence in the HCF samples was assigned to *Bacteroides* (20.20%), *Megamonas* (6.81%), *Enterobacter* (5.31%), and *Faecalibacterium* (4.86%) (**Figure 2B**). For the SLEV group, *Prevotella* accounted for 18.47% of the bacterial sequence, followed by *Bacteroides* (10.99%), *Megamonas* (6.17%), *Lactobacillus* (5.47%), and *Phascolarctobacterium* (4.33%). For the HCV group, 75.59% of the bacterial sequence was assigned to *Lactobacillus*, followed by *Prevotella* (9.35%), *Gardnerella* (9.29%), and *Atopobium* (1.90%) (**Figure 2B**).

### SLE patients had different bacterial phyla and genera

**Figure 3:**
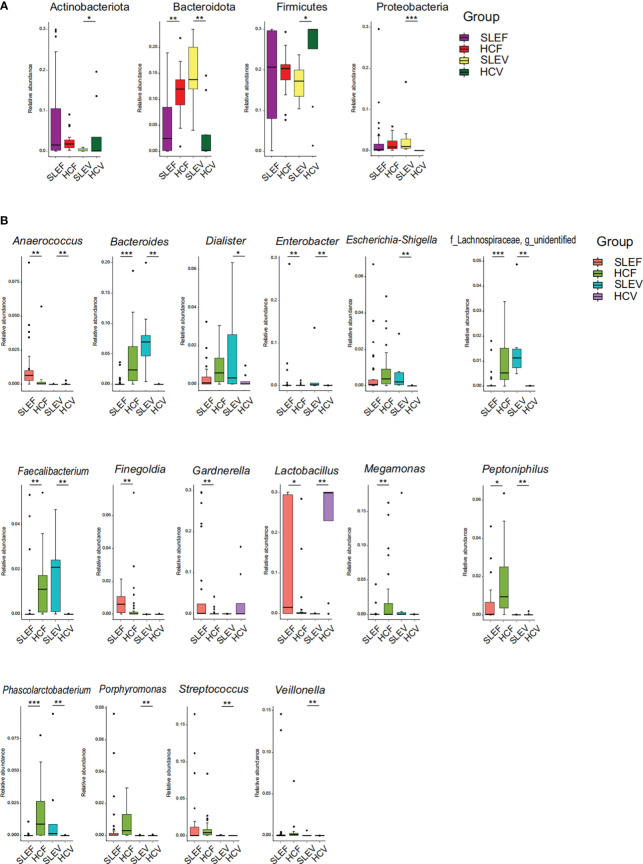
Bacterial taxons that were differentially abundant between SLE patients and controls. **(A)** Bacterial phyla significantly differed in SLE patients compared with those in controls. **(B)** Bacterial genera significantly differed in SLE patients compared with those in controls. The P-value was calculated using the Wilcoxon rank-sum test and adjusted using the Benjamini and Hochberg FDR. *FDR <0.05; **FDR <0.01; ***FDR <0.001.

When the bacterial phyla were compared among the fecal samples, there was a depletion of Bacteroidota in the SLE patients (FDR=0.005; **Figure 3A**). When the bacterial phyla in the vaginal samples were compared, three bacterial phyla showing significant differences between SLEV and HCV groups, such as Actinobacteria and Firmicutes, sharply reduced in the SLEV samples, whereas Proteobacteria significantly increased in SLEV samples (FDR of <0.05; **Figure 3A**).

The bacterial genera accounting for more than 1% of the total abundance were compared. A total of 11 genera showed a significant difference between SLEF and HCF (FDR of <0.05; **Figure 3B**). Five bacterial genera, *Anaerococcus*, *Finegoldia*, *Gardnerella*, *Lactobacillus*, and *Peptoniphilus*, were enriched in the SLEF samples. However, six bacterial genera, *Bacteroides*, *Enterobacter*, *Faecalibacterium*, *Megamonas*, *Phascolarctobacterium*, and an unidentified genus of the bacterial Lachnospiraceae family, were depleted in the SLEF samples.

Thirteen bacterial genera (more than 1% of the total abundance) differed in SLEV samples (FDR of <0.05, **Figure 3B**). Almost all of them increased in SLEV, except for *Lactobacillus*. To be specific, the SLEV samples had higher abundances of *Anaerococcus*, *Bacteroides*, *Dialister*, *Enterobacter*, *Escherichia-Shigella*, *Faecalibacterium*, *Peptoniphilus*, *Phascolarctobacterium*, *Porphyromonas*, *Streptococcus*, *Veillonella*, and an unidentified bacterial genus of the Lachnospiraceae family.

**Figure 4:**
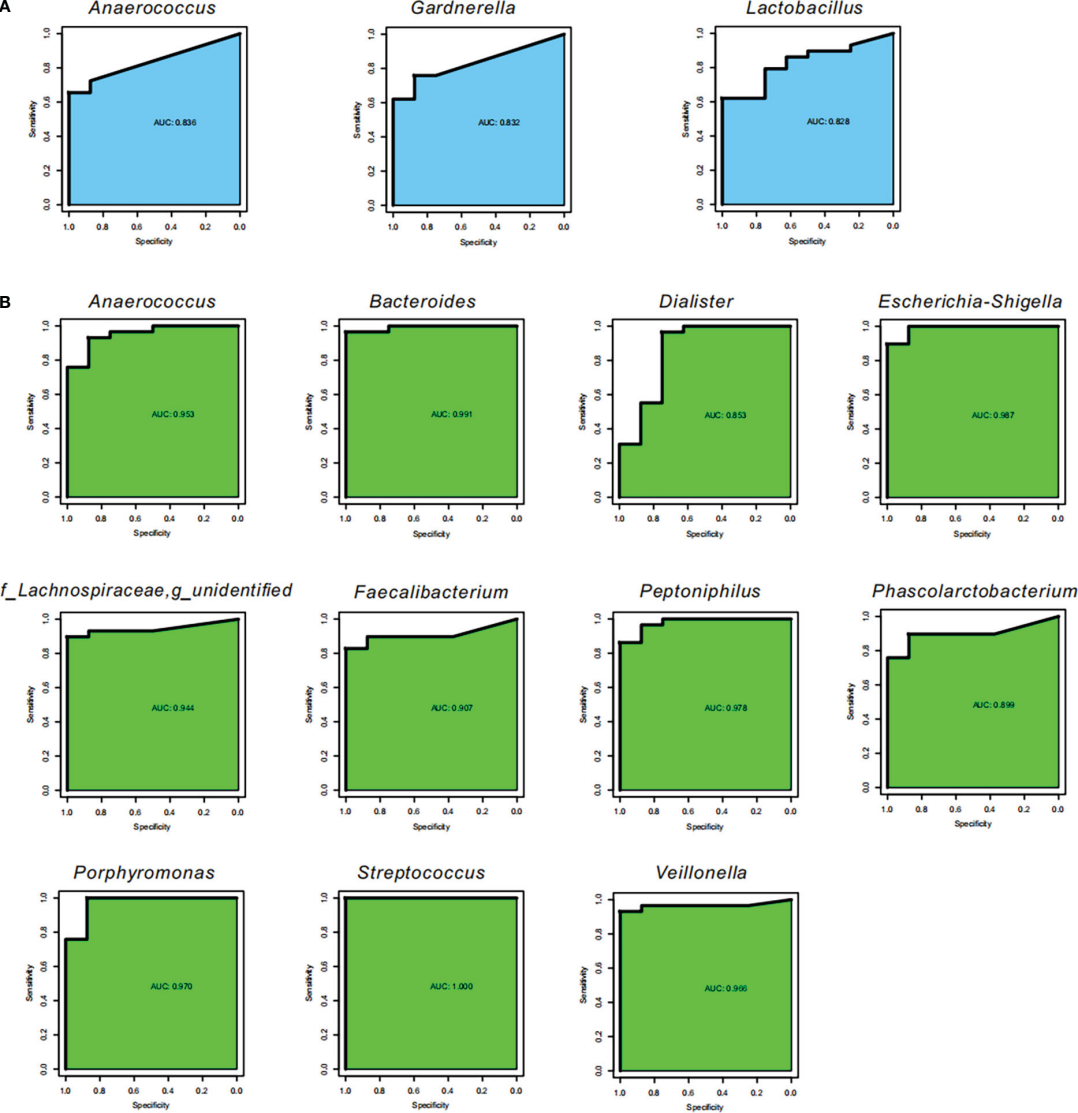
Potential biomarkers. Receiver operating characteristic curve (ROC) for the validation of microbial classification of control and SLE patients. Sensitivity is on the y-axis and specificity is on the x-axis. The area under the curve (AUC) is blue for fecal samples **(A)**, and green for vaginal samples **(B)**.

We also performed ROC to explore potential biomarkers for SLE and noticed that three bacterial genera, *Anaerococcus*, *Gardnerella*, and *Lactobacillus*, could be considered as biomarkers for identifying SLE from HC in feces (AUC=0.828−0.836; **Figure 4A**), which demonstrated that the three bacterial genera had an 82.80−83.60% chance of correctly distinguishing the SLE patients from the HC. In the vagina, 11 bacterial genera, *Bacteroides*, *Escherichia-Shigella*, and *Streptococcus*, could be listed as biomarkers for identifying SLE (AUC=0.899−1; **Figure 4B**), which demonstrated that the three bacterial genera had a 89.90−100.00% chance of correctly distinguishing the SLE patients from the HC.

### Microbiomes in feces and the vagina were associated

**Figure 5:**
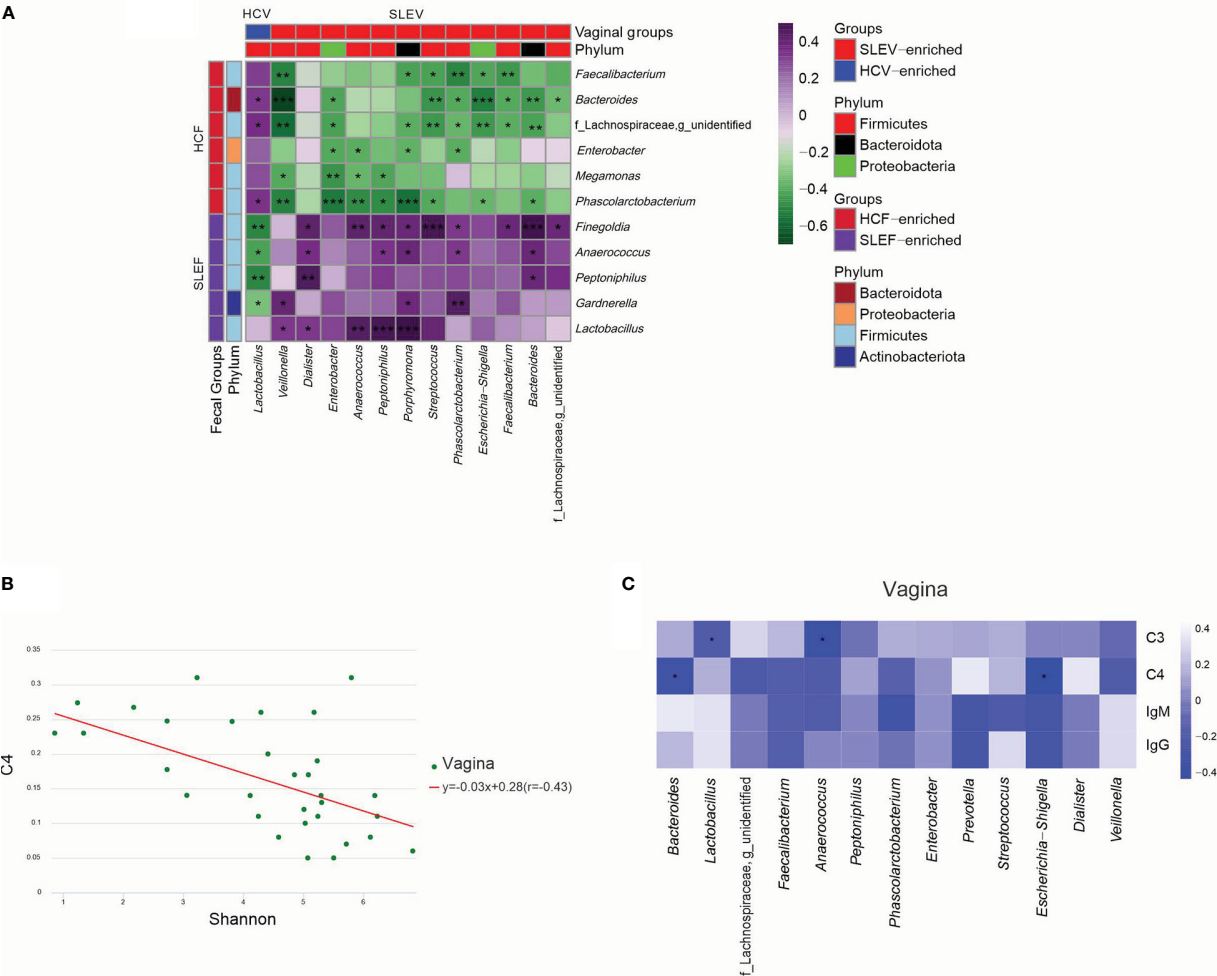
Associations of the microbiome. **(A)** Pearson correlation analysis was performed between the bacterial genera that differed in the feces of SLE patients and bacterial genera that differed in the vaginas of patients. **(B)** Pearson correlation analysis was performed between the Shannon index and serum C4 in the vaginas of SLE patients. **(C)** Pearson correlation analysis was performed between the bacterial genera that differed in the vaginas of patients and their immunological features. The correlation of two variables with values of |*r*|>0.3 and *P <*0.05 are displayed. *P <0.05; **P <0.01; ***P <0.001.

As mentioned above, we found that several fecal differential genera were observed between the groups of SLE and HC, while other vaginal differential genera were found between the two groups. Pearson correlation analysis demonstrated significant correlations between these fecal and vaginal differential genera (**Figure 5A**). For example, *Lactobacillus* in SLEV was positively correlated with *Bacteroides* in SLEF, whereas *Lactobacillus* in SLEV was negatively correlated with *Gardnerella* in SLEF (r>0.30, P<0.05). Notably, *Lactobacillus* in SLEV was not correlated with *Lactobacillus* in SLEF (r=0.002, P=0.990). *Escherichia−Shigella*, which increased in the SLEV samples, was negatively correlated with *Bacteroides* and *Faecalibacterium*, which decreased in SLEF (r>0.30, P<0.05).

### Immunological profiles in SLE patients were associated with the vaginal microbiome

When Pearson correlation analysis was performed to demonstrate the associations between the microbiome and immunological profiles, we found that the Shannon index in the vaginas of SLE patients was negatively associated with serum C4 (r=-0.430, P=0.041; **Figure 5B**). Next, we performed Pearson correlation analysis between the differed bacterial genera in the feces and vaginas of patients and their differed immunological features, and no significant correlation was found in the fecal microbiome (P>0.05), while significant correlations were found in the vaginal microbiome (P<0.05; **Figure 5C**). For example, serum C4 was negatively correlated with *Bacteroides* and *Escherichia−Shigella* in the vaginas of patients (P<0.05; **Figure 5C**).

## Discussion

On one hand, we comprehensively characterized the gut and the vaginal microbiomes and their associations in SLE patients. On the other hand, we found that the microbiome in patients’ vaginas can better predict the disease and disordered immunological profiles than those in patients’ feces.

Our present study found that the β diversities of the gut and vagina were significantly altered in SLE patients. The alteration of the gut microbiome community in SLE patients is in line with some previous studies (6–13). As far as whether the microbiomes in the gut and other body niches alter simultaneously, the findings in previous studies are not consistent. Liu et al. found that both the gut and saliva microbiomes altered simultaneously in SLE patients (10), while James et al. and van der Meulen et al. found that only plasma or the oral cavity, rather than the gut, had a distinct microbial community with respect to the controls (5, 16). The inconsistent findings might be due to the fact that the study participants in the previous pilot studies and ours consisted of people from different countries and locations or the small numbers of participants recruited to these studies, which decreases the chance of achieving a precise result (31).

Compared with the controls, bacterial richness and diversity in the guts and vaginas of SLE patients showed opposite changes. Similar findings were not observed in previous studies, which examined bacterial microbiomes in multi-body niches in SLE patients. It is reported that the bacterial diversity in human feces tends to decrease in unhealthy conditions (32), including SLE (10, 11). However, the bacterial diversity in the human vagina tends to increase in unhealthy conditions, such as bacterial vaginosis (33), and in preterm women (34). Thus, the alterations in the microbial diversity in the gut and vagina in our study indicate that these two body niches had unhealthy microbiomes.

Notably, the differed bacterial diversity was only observed in the SLE vagina. In addition, the shared ASVs between SLEV and HCV were less than those between SLEF and HCF, and the bacterial genera that can be considered as biomarkers for identifying SLE subjects from controls in the vagina were more than those in the gut. These findings suggest that the dysbiosis in the vaginas of patients was more obvious than that in their gut.

In the feces of SLE patients, we demonstrated that *Lactobacillus* sharply increased and served as an identifying biomarker. As several previous studies have reported that feeding *Lactobacillus* spp. to lupus mice can impact lupus progression by enhancing immunoregulation (35), preventing vascular disorders (36), or exerting anti-inflammatory effects (37), the enrichment of *Lactobacillus* in patients’ guts might be a protective response. Unlike in the gut, *Lactobacillus* sharply declined in patients’ vaginas. A lack of *Lactobacillus* is one of characteristics of bacterial vaginosis (38). Although SLE patients with bacterial vaginosis were excluded from our study, the patients presented a vaginosis-like vaginal microbiome, which indicates that patients’ vaginas were in an unhealthy condition. A further study should follow the patients for several years to see whether the women with vaginal dysbiosis are prone to vaginitis.

We observed that several bacterial genera altered in patients’ gut responded to several bacterial genera that altered in their vagina. *Gardnerella*, a bacteria involved in vaginitis and ulcerative colitis (39–41), displayed higher abundance in the guts of SLE patients. Additionally, *Gardnerella* in the guts of patients was negatively associated with their vaginal *Lactobacillus*. These findings suggest that microbiomes in the gut and vagina may mediate each other. Further study is needed to explore their interaction effects.

*Faecalibacterium*, an anaerobic bacterium, is one of the most important butyrate-producing bacteria and has been considered as a bioindicator of health in the human gut (42), where it decreases in SLE patients (9, 43). This depletion might play a pathological role in the onset and progression of SLE. Moreover, it was responsible for the increase of *Streptococcus* and *Escherichia−Shigella* in their vagina. *Streptococcus* and *Escherichia−Shigella* are important pathogens and emerging causes of unhealthy conditions, such as vaginitis (44), miscarriage, and stillbirth (45). Therefore, the decrease of *Faecalibacterium* in patients’ guts and the increase of *Streptococcus* and *Escherichia−Shigella* in their vaginas and their negative associations might indicate that modulations of the promotion of growth of *Faecalibacterium* in patients’ guts may impact SLE progression by inhibiting the growth of *Streptococcus* and *Escherichia−Shigella* in patients’ vaginas.

Although it is known that imbalances within the gut microbiomes of SLE patients contribute to immunity (46), we only observed an association between the vaginal microbiome with patients’ immunological profiles. To be specific, the bacterial diversity in patients’ vaginas negatively responded to their serum C4. As the patients were characterized by a higher bacterial diversity and a lower serum C4 compared with controls, their negative association implies that the interventions that can lower the bacterial diversity in patients’ vaginas may regulate their immunological disorder. Meanwhile, we noticed that only the bacterial genera that were altered in patients’ vaginas were associated with their immunological features, e.g., *Escherichia−Shigella* was negatively associated with patient’s serum C4. *Escherichia−Shigella* is not only a primary cause of urinary tract infection but can also can invade vaginal cells and play a potential role in infection 47. The negative association between vaginal *Escherichia−Shigella* and serum C4 suggests that the elimination of vaginal *Escherichia−Shigella* can restore disordered immunity in SLE patients.

There are several limitations to our present study. First, we recruited SLE women rather than men, thus it is impossible to conclude whether the reproductive microbiome in men altered and determine the gender role of the microbiome in SLE. Second, vaginal samples were collected from patients not affected by vaginitis, and we cannot reveal the correlations between the vaginal microbiome and vaginal infection in patients. Third, this is a pilot study without animal models, the cause-effect relationships between gut and vagina microbiome, and between vaginal microbiome and immunity, remain unclear. Further study using animal models is necessary to explore the cause-effect relationships.

In summary, our present study revealed that SLE women had distinct microbiomes in their gut and vagina, and these microbiomes interact with each other. Compared with the gut microbiome, it seems that the vaginal microbiome in patients plays a bigger role in the disease. Further study should explore how the microbiomes in the guts and vaginas of patients interact and how the vaginal microbiome regulates patients’ immunity.

## Data availability statement

Sequencing data from this study have been deposited in the GenBank Sequence Read Archive under accession number PRJNA904086 (https://www.ncbi.nlm.nih.gov/bioproject/PRJNA904086).

## Ethics statement

The studies involving human participants were reviewed and approved by Lishui Second People’s Hospital (Zhejiang, China) and the First Affiliated Hospital, School of Medicine, Zhejiang University. The patients/participants provided their written informed consent to participate in this study.

## Author contributions

ZL and RK conceived and designed the experiments. ZL, YC, JG, WL, XY, XH, LS, XL, and RK performed the experiments. ZL, YC, LS, and XL analyzed the data. ZL, YC, and RK wrote the paper and edited the manuscript. All authors contributed to the article and approved the submitted version.

## Supplementary material

Supplementary Figure 1 Bacterial composition between the medication users and non-users. **(A)** Comparison between azathioprine users and non-users. **(B)** Comparison between hydroxychloroquine users and non-users. **(C)** Comparison between prednisolone users and non-users. PCoA based on Bray−Curtis distances ASV level. The 95% confidence ellipse is drawn for each group. Permutational multivariate analysis of variance (PERMANOVA) was performed for statistical comparisons of samples in the two groups. The *P* value was adjusted using the Benjamini and Hochberg false discovery rate (FDR). HCF, HC feces; HCV, HC vagina; SLEF, systemic lupus erythematosus feces; SLEV, systemic lupus erythematosus vagina.
