# Supplementary figures and images for: Alterations of the fecal and vaginal microbiomes in patients with systemic lupus erythematosus and their associations with immunological profiles
Source: Front Immunol. 2023 Mar 10;14:1135861. doi: 10.3389/fimmu.2023.1135861 (PMC10036376; doi:10.3389/fimmu.2023.1135861)

A

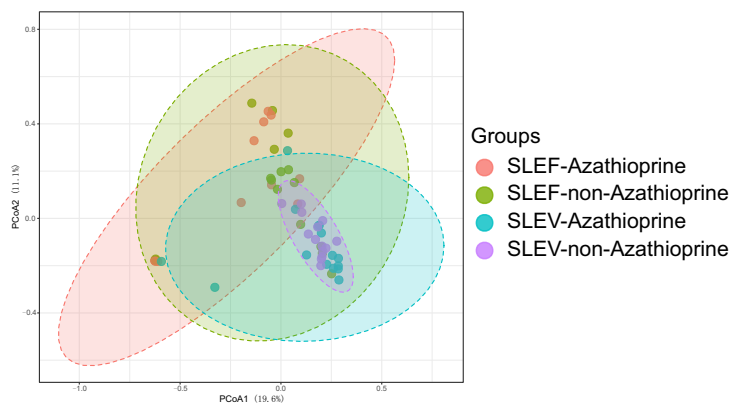

B

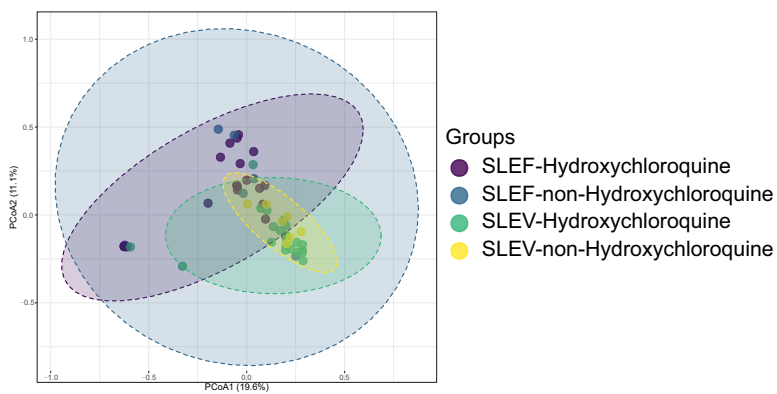

C

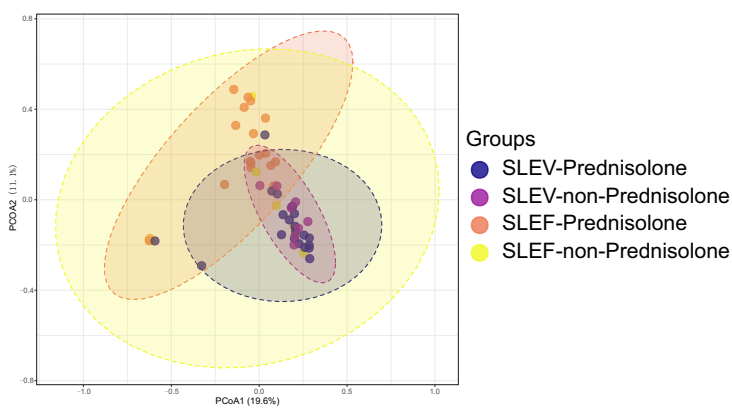

Supplement: Supplementary Figure 1 — Bacterial composition between the medication users and non-users. (A) Comparison between azathioprine users and non-users. (B) Comparison between hydroxychloroquine users and non-users. (C) Comparison between prednisolone users and non-users. PCoA based on Bray−Curtis distances ASV level. The 95% confidence ellipse is drawn for each group. Permutational multivariate analysis of variance (PERMANOVA) was performed for statistical comparisons of samples in the two groups. The P value was adjusted using the Benjamini and Hochberg false discovery rate (FDR). HCF, HC feces; HCV, HC vagina; SLEF, systemic lupus erythematosus feces; SLEV, systemic lupus erythematosus vagina. [file Image_1.pdf]
